# Visual-Attention-Based Neighbor Selection for Artificial-Potential-Field UAV Formation Control

**DOI:** 10.3390/s26144479

**Published:** 2026-07-14

**Authors:** Miao Yu

**Affiliations:** School of Mathematics and Statistics, Xidian University, Xi’an 710071, China; myu@xidian.edu.cn

**Keywords:** formation control, distributed artificial potential field, visual attention mechanism, neighbor selection

## Abstract

The development of Unmanned Aerial Vehicle (UAV) formation technology is rapid, and formation flying under complex conditions has also received more attention. However, neighbor selection without reliable radio communication remains challenging because fixed-radius or fixed-topology methods may process redundant neighbor states. Based on this, this paper designs two visual-attention-based neighbor-selection algorithms, namely Visual Attention Potential Field (VAPF) and Cluster Visual Attention Potential Field (CVAPF). Both algorithms use a zoom-lens visual attention rule to retain informative neighbors before the artificial-potential-field control input is evaluated. The algorithm VAPF selects informative UAV-level neighbors for the APF controller and supports aggregation behavior in simulation. Algorithm CVAPF extends the selection rule to clusters through a dual layer communication architecture, which improves synchronization while gathering formations. In the tested redundant sensing scene, BOIDS and Optimized-flocking process 43.38 and 15.20 neighbors on average, whereas VAPF and CVAPF reduce the values to 6.31 and 3.60 while preserving the formation behavior observed in the simulations.

## 1. Introduction

Unmanned Aerial Vehicle (UAV) formation control is important for cooperative sensing and operation in complex environments [1,2,3,4]. Representative applications include photogrammetry [5], humanitarian response [6], and reconnaissance or surveillance [7]. At the algorithmic level, multi-agent consensus provides a basic framework for coordinating many UAVs through local state exchange [8,9]. In this paper, the target is to support APF-driven formation behavior while reducing the number of neighbor states processed by each UAV when reliable radio communication is unavailable.

Formation control in complex environments has been studied from distributed control, multi-UAV coordination, and real-flight perspectives [10,11,12,13]. However, these settings are difficult when communication infrastructure is weak or unavailable. Vision-assisted sensing offers a practical way to estimate neighboring UAV states and support autonomous formation flight; for example, 3D UAV pose estimation can provide target position and attitude information [14], and dense-environment flight demonstrates the value of distributed local control [15]. For this reason, the neighbor-selection rule becomes a central design issue: a UAV should process enough visual neighbors for APF-driven aggregation and synchronization, but avoid processing redundant observations that do not improve the controller.

The research objective of this paper is to design a visual-attention-based neighbor-selection topology for artificial-potential-field UAV formation control. The central research question is whether a UAV can suppress angularly redundant visual neighbors before controller evaluation while still retaining the visually informative neighbors needed for aggregation and synchronization. The main challenges are therefore summarized as follows: (1) selecting informative neighbors without processing all visible UAVs, (2) maintaining formation behavior when radio-based fixed topologies are unavailable, and (3) improving motion order after local aggregation without requiring every UAV to process all visible neighbors.

To address these challenges, this paper designs a visual attention neighbor-selection topology for APF-based formation control. The significance of this design is that each UAV can retain visually informative neighbors for aggregation and synchronization while reducing the number of neighbor states that must be processed. Under the Visual Attention Potential Field (VAPF) algorithm, UAVs select neighbors through visual attention, and the APF controller then uses the selected neighbor states to drive formation motion. Because pure aggregation can still lead to locally disordered trajectories after UAVs approach one another, this paper further introduces the Cluster Visual Attention Potential Field (CVAPF) algorithm. CVAPF uses cluster-level visual attention, a two-layer communication architecture, and cluster merging to improve synchronization without requiring every UAV to process all visible neighbors.

The contribution of this paper is as follows.

(1) A leader flight planning model is designed so that UAV.1 tracks preset velocities along a reference trajectory and provides a moving reference for the formation.
(2) Two attention-based neighbor-selection algorithms are developed for APF-based formation control. VAPF uses zoom-lens visual attention to select informative UAV-level neighbors before APF control evaluation. CVAPF extends this idea to cluster-level attention and uses a two-layer communication architecture with cluster merging to reduce redundant neighbor processing at the cluster level.
(3) Geometric boundary conditions are discussed for collision avoidance and communication maintenance under the stated assumptions. These conditions are used as practical design constraints for the attention-generated topology rather than as a full stability proof.

Modeling level: The proposed algorithms are developed at the formation control layer, where each UAV updates its motion from relative neighbor states selected by the visual attention rule. The UAV motion is therefore represented by a kinematic point-mass model, and visible neighbor positions and velocities are treated as available state inputs to the controller. This abstraction allows the analysis to concentrate on the central question of this paper: how the attention-generated topology changes the number and relevance of neighbor states processed before the artificial-potential-field input is evaluated. Deployment-level sensing errors, communication imperfections, and low-level multirotor dynamics are left for future implementation-oriented studies.

The rest of this paper is organized as follows. Section 2 reviews related work. Section 3 introduces the preliminaries. Section 4 presents the proposed method, including leader flight planning, VAPF, CVAPF, and the boundary conditions. Section 5 reports simulation results and discussion, including the comparative neighbor processing and attention ratio analyses. Section 6 concludes the paper.

## 2. Related Work

This section summarizes the literature most directly related to the proposed neighbor-selection topology. It first reviews flocking and artificial-potential-field methods that motivate the control component, then reviews metric, topology-based, and visual attention neighbor-selection models that motivate the perception component.

Flocking and artificial-potential-field models provide the most relevant methodological basis for this work. Classical BOIDS (bird-oid) flocking [16] and visual movement models [17,18] show that collective motion can emerge from local rules. Optimized-flocking of autonomous drones further shows that collision avoidance, velocity alignment, sensing delay, and motion constraints must be considered together in real aerial robot swarms [19]. In parallel with these flocking models, artificial-potential-field (APF) methods provide a controller-level way to convert relative position or target information into attractive and repulsive virtual forces. This idea has been widely used for autonomous trajectory generation, local navigation, and obstacle avoidance [19,20,21]. APF-based methods may suffer from local minima when target attraction and obstacle repulsion cancel in obstacle-rich planning environments. We therefore treat this issue as an APF limitation outside the present focus on inter-UAV neighbor selection before potential-field evaluation. These studies justify the potential-field controller used in this paper, but they do not directly solve how a vision-based UAV should suppress angularly redundant neighbors before the controller is evaluated.

The remaining gap is how to choose interaction neighbors when the topology is generated from visual perception rather than from radio links or a fixed metric neighborhood. Metric models select neighbors within a distance threshold [22,23], and UAV formations often use predefined or directly constructed communication topologies [24]. However, these rules can become inefficient or unavailable when radio communication is not reliable. Visual models provide another route: visual collective-behavior frameworks [25], studies of individual and collective vision [26], and zoom-lens/spotlight attention models [27] show that agents can process only selected visual sectors. This paper therefore relates most closely to BOIDS and optimized drone flocking [16,19] on the control side, and to visual attention models [27] on the perception side. The specific gap addressed here is the lack of a neighbor-selection topology that combines potential-field formation control with attention-based suppression of redundant visual neighbors.

Recent large-scale aerial-swarm planning and robot-swarm assembly studies further show that scalability, resilience, and low per-agent processing load are central requirements for practical swarm systems [21,28,29]. Existing orderly-flight strategies include preset formation assignment [20] and trajectory planning for each UAV [30,31]. These studies are complementary to the present work: they address formation planning, assignment, or swarm assembly, whereas this paper focuses on the visual neighbor-selection topology used before the potential-field controller is evaluated.

## 3. Preliminaries

This section introduces the mathematical notation and basic mechanisms used by the proposed algorithms. The goal is to make the later leader-planning, VAPF, and CVAPF equations readable without repeatedly redefining common graph, potential-field, and visual attention concepts.

The section is organized into three parts. First, graph notation defines the neighbor sets used by the distributed topology; second, the artificial-potential-field components define attraction, velocity alignment, and repulsion; third, the visual attention rule explains how redundant visual neighbors are removed before the controller is evaluated.

### 3.1. Knowledge of Graph Theory

Graph notation is used to describe which UAV states are processed by each distributed controller. In this paper, the graph is not assumed to be a fixed radio topology; it can be generated by the visual attention selection rule. Distributed communication in formation requires the establishment of communication topology graph G. Consider a directed communication graph G=(V,E,A), $V=\left\{1,2,3....,n\right\}$ as a set of nodes, E⊂V×V is set of edges (directed edges), and adjacency matrix $A={\left(a_{ij}\right)}_{n\times n}$ with a non-negative element (this paper does not consider the weight of each edge). $N_{i}=\left\{j\in V|(j,i)\in E,i\neq j\right\}$ is the set of parent nodes of *i*. $∥.∥$ represents the Euclidean norm.

### 3.2. Artificial Potential Field

This subsection defines the potential-field components used after VAPF and CVAPF have selected their neighbors and explains why these components are arranged in distance-dependent regions. Existing APF-based navigation and obstacle-avoidance studies commonly construct control inputs from attractive or repulsive virtual potentials [32,33,34]. In this paper, the same gradient-based idea is used for inter-UAV formation interaction: selected neighboring UAVs act as moving potential-field sources, and the follower UAV evaluates only the sources retained by the visual attention topology.

**Figure 1:**
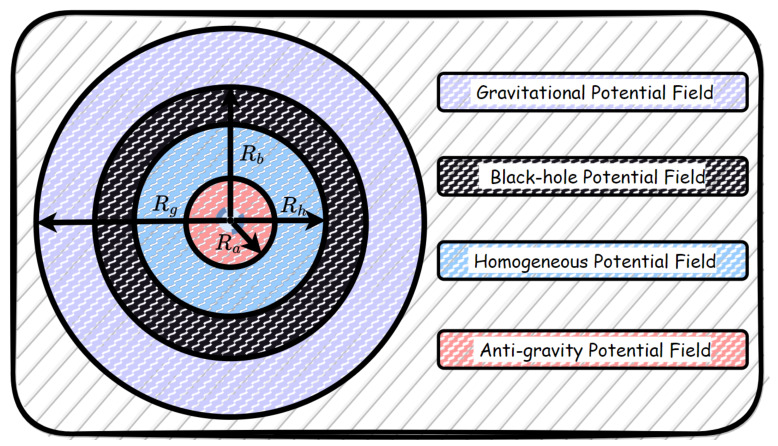
Artificial potential field generated by an individual UAV. The arrows indicate radial distance directions from the source.

The four potential-field regions in Figure 1 are designed to reproduce the main local interaction roles used in flocking and formation control. At long range, the gravitational field provides cohesion by attracting separated UAVs toward selected neighbors. At intermediate range, the black-hole field acts as a shaped attraction term that strengthens gathering before the UAV reaches the velocity alignment zone; it should be understood as a designed transition potential rather than as a separate physical phenomenon. Near the desired interaction range, the homogeneous field penalizes velocity differences and therefore plays the role of flocking alignment. At very short range, the anti-gravity field dominates the other terms so that separation and collision avoidance have priority over aggregation.

The following equations define these regions in a continuous order from long-range attraction to short-range repulsion. The first region is the long-range cohesion region: its quadratic potential produces a force proportional to the relative displacement, so a selected neighbor pulls the UAV toward the formation when the distance is large. When $∥x-x_{goal}∥\in \left[R_{b},R_{g}\right]$, the gravitational potential field $\Upsilon_{g}\left(x\right)$ is represented as

(1) $$\Upsilon_{g}\left(x\right)=\frac{1}{2}k_{g}{∥x-x_{goal}∥}^{2},$$

where $k_{g}$ is the coefficient related to the strength of the gravitational potential field; $x_{goal}$ represents the position of the potential field source; $R_{b}$ represents the radius of the black-hole potential field range; and $R_{g}$ represents the radius of the gravitational potential field range. The corresponding force $f_{g}\left(x\right)$ is expressed as

(2) $$f_{g}\left(x\right)=-▽\Upsilon_{g}\left(x\right)=-k_{g}\left(x-x_{goal}\right).$$

Thus, the role of this region is only cohesion; velocity matching and collision avoidance are activated by the inner regions below.

The second region is a transition attraction region. Its purpose is to keep the attractive force active while preventing an abrupt switch between long-range cohesion and the inner alignment region. When $∥x-x_{goal}∥\in \left[R_{h},R_{b}\right],$ the black-hole potential field $\Upsilon_{b}\left(x\right)$ is represented as

(3) $$\Upsilon_{b}\left(x\right)=-\frac{1}{2}k_{b}{\left(R_{b}-∥x-x_{goal}∥\right)}^{2}+\frac{1}{2}k_{g}{∥x-x_{goal}∥}^{2},$$

where $k_{b}$ is the coefficient related to the strength of the black-hole potential field; $R_{h}$ represents the radius of the homogeneous potential field range. The corresponding force $f_{b}\left(x\right)$ is expressed as

(4) $$\begin{array}{c} f_{b}\left(x\right)=-▽\Upsilon_{b}\left(x\right)\\ \;\;=-k_{b}\left[R_{b}\frac{x-x_{goal}}{∥x-x_{goal}∥}-\left(x-x_{goal}\right)\right]-k_{g}\left(x-x_{goal}\right). \end{array}$$

This shaped term is named the black-hole field in this manuscript because it increases the tendency of separated UAVs to gather before they enter the alignment range; it is a designed APF component, not a physical black-hole model.

After a neighbor enters the desired interaction range, position attraction is no longer the only objective. The homogeneous field is defined on velocity difference so that nearby UAVs reduce relative speed and move coherently. When $\;\;∥x-x_{goal}∥\in \left[R_{a},R_{h}\right],$ the homogeneous potential field $\Upsilon_{h}\left(v\right)$ is represented as

(5) $$\begin{array}{c} \Upsilon_{h}\left(v\right)=\frac{1}{2}k_{h}{∥v-v_{goal}∥}^{2}, \end{array}$$

where $k_{h}$ is the coefficient related to the strength of the homogeneous potential field; $v_{goal}$ represents the velocity of the potential field source; and $R_{a}$ represents the radius of the anti-gravity potential field range. The corresponding force $f_{h}\left(v\right)$ is expressed as

(6) $$\begin{array}{c} f_{h}\left(v\right)=-▽\Upsilon_{h}\left(v\right)=-k_{h}\left(v-v_{goal}\right). \end{array}$$

This term corresponds to the alignment rule in flocking models: the UAV does not simply move closer to the neighbor, but adjusts its velocity toward the neighbor velocity.

The innermost region is reserved for safety. When the distance is below $R_{a}$, the repulsive potential grows rapidly as the distance decreases, so short-range separation dominates the attraction and alignment terms. When $\;\;∥x-x_{goal}∥\in \left(0,R_{a}\right]$, the anti-gravity potential field $\Upsilon_{a}\left(x\right)$ is represented as

(7) $$\begin{array}{c} \Upsilon_{a}\left(x\right)=\frac{1}{2}k_{a}{\left(\frac{1}{∥x-x_{goal}∥}-\frac{1}{R_{a}}\right)}^{2}, \end{array}$$

where $k_{a}$ is the coefficient related to the strength of the anti-gravity potential field. The corresponding force $f_{a}\left(x\right)$ is expressed as

(8) $$\begin{array}{c} f_{a}\left(x\right)=-▽\Upsilon_{a}\left(x\right)\\ \;\;=k_{a}\left\{\frac{1}{{\left(x-x_{goal}\right)}^{3}}-\frac{1}{R_{a}∥x-x_{goal}∥\left(x-x_{goal}\right)}\right\}. \end{array}$$

The above APF design does not claim to eliminate the general local minimum problem of APF path planning. Local minima usually arise in environments with static or dynamic obstacles, where target attraction and obstacle repulsion can cancel and trap the robot before it reaches the goal. The present manuscript does not model external obstacles or global path planning; it studies how visual attention selects inter-UAV neighbors before the formation control input is evaluated. Therefore, the local minimum issue is treated as an APF limitation outside the current obstacle-free neighbor-selection setting.

### 3.3. Visual Attention Selects Neighbors

This subsection defines the visual attention rule used to construct the neighbor set before the potential-field controller is evaluated. The rule keeps visually informative targets and masks angularly redundant targets that are covered by already selected perceived cones.

**Figure 2:**
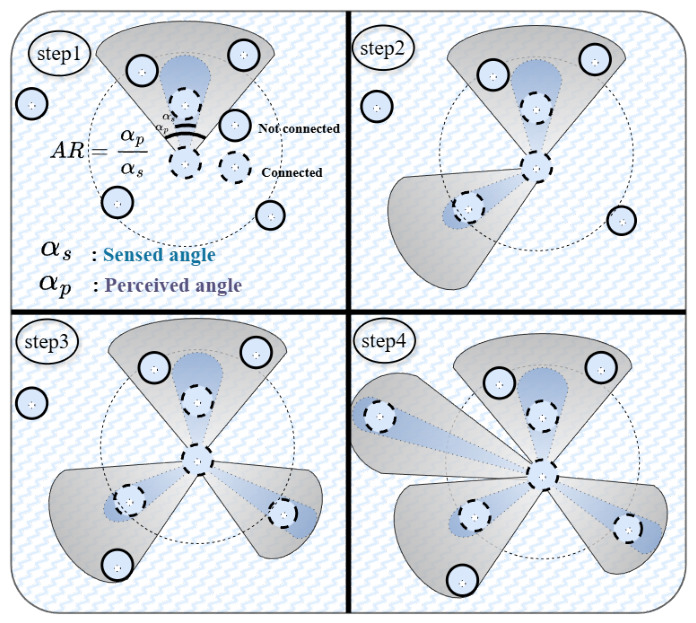
Visual attention mechanism for selecting neighboring nodes. Light-blue sectors denote sensed angles, gray sectors denote perceived defocusing angles, black dashed outlines mark selected neighboring UAVs, and the four panels show the sequential selection process.

The zoom-lens model is an attention mechanism model that originates from the principle of camera imaging. When the camera focuses on an object, it also defocus around the object. Similar to the human eye tracking moving targets, this attention model is consistent with biological intuition. Focusing can help the eye capture the state of objects, and defocusing can filter out other unimportant information. The Sensed angle and Perceived angle in this paper is a conical field of view in 3D space. Sensed angle is the range we focus on, which precisely wraps around the target; Perceived angle is the range within which we defocus, which means that other targets within that range will not be focused (Perceived angle = AR· Sensed angle). Therefore, in the UAV formation, each UAV selects neighbors according to the following steps (refer to Figure 2): Step 1: Select the UAV with the largest Sensed angle as the neighbor node, while defocusing within the Perceived angle. Step 2: Select UAVs outside of the Perceived angle. Step 3: Repeat the above process until no UAV can be selected. Step 4: Output the selected UAVs as the neighbor set $N_{i}$ used by the potential-field controller. In Figure 2, this step represents the final retained visual targets after redundant targets inside perceived cones have been discarded. This attention mechanism has some properties in cluster systems.

(1) The average distance of individual nearest neighbors in a cluster is called cluster sparsity (CP); the total number of individuals in a cluster is called cluster size (CS). When AR is a fixed value, as CP and CS increase, the number of neighbors chosen by individuals will also increase. We call this phenomenon scale-dependence. When $AR_{i}=distance_{i}$ (distance from individual to node i), as CP and CS increase, the number of neighbors selected by individuals will saturate within a certain range. We call this phenomenon scale-free, which is a method that conforms to biological visual processing information: biological processing information has boundaries.
(2) Individuals tend to focus on isolated nodes or parts with low CP. This phenomenon is consistent with biological behavior, with less attention given to individuals in clusters and more attention given to isolated individuals, just as wolves tend to prey on sheep that leave the flock.

## 4. Proposed Method

This section presents the proposed leader-planning, VAPF, and CVAPF methods. The objective is to show how visual attention neighbor selection is coupled with artificial-potential-field control at both the individual-UAV and cluster levels.

The section proceeds as follows. First, a leader trajectory-tracking model is introduced to provide the formation reference. Second, VAPF is defined as an individual-level attention-potential-field method. Third, CVAPF extends the same idea to clusters through cluster attention, two-layer communication, and merging. Finally, geometric boundary conditions are discussed as practical constraints for collision avoidance and communication maintenance.

(1) Develop a virtual UAV algorithm for the formation leader, which can manage the speed and trajectory of the formation. (*Subsection A*)
(2) Propose two visual-attention-based neighbor-selection algorithms (VAPF and CVAPF) for generating the APF communication topology. (*Subsections B and C*)
(3) Calculate the corresponding boundary conditions used as practical constraints for the attention-generated neighbor topology. (*Subsection D*)

### 4.1. Leader Flight Planning

The leader-planning model defines the moving reference followed by the rest of the formation. It is not intended to solve global path planning; instead, it converts a given reference trajectory and desired velocity sequence into the leader’s discrete-time motion. Starting from the formation leader (UAV.1) and ending at the target position, we generate a continuous trajectory, denoted as *L* (we will not discuss how to plan the trajectory). Divide the trajectory into $n_{l}$ segments to obtain $n_{l}+1$ points, denoted as ${\left\{l^{\left(k\right)}\right\}}_{k=1}^{n_{l}+1}$. In the $k_{l}$-th segment of the trajectory, we preset the expected velocity of UAV.1 as $v_{L}^{\left(k+1\right)}$. We represent UAV.1 motion modeling as

(9) $$\begin{array}{cc} x_{1}\left[n+1\right] & =x_{1}\left[n\right]+\min\left\{∥v_{1}\left[n\right]∥,∥v_{max}∥\right\}\frac{v_{1}\left[n\right]}{∥v_{1}\left[n\right]∥}\Delta t, \end{array}$$

(10) $$\begin{array}{cc} v_{1}\left[n+1\right] & =v_{1}\left[n\right]+\min\left\{∥u_{1}\left[n\right]∥,∥u_{max}∥\right\}\frac{u_{1}\left[n\right]}{∥u_{1}\left[n\right]∥}\Delta t, \end{array}$$

(11) $$\begin{array}{cc} u_{1}\left[n+1\right] & =\sum_{k=1}^{n_{l}}\left\{k_{L}^{\left(k+1\right)}\left(v_{1}\left[n\right]-v_{L}^{\left(k+1\right)}\right)\;\tau \left(∥\left(l^{\left(k+1\right)}-x_{1}\left[n\right]\right)\left(x_{1}\left[n\right]-l^{\left(k\right)}\right)∥\right)\right\}, \end{array}$$

where $x_{1}\left[n\right]$ represents the position of UAV.1; $v_{1}\left[n\right]$ represents the speed of UAV.1; $∥v_{max}∥$ represents the maximum speed of the UAVs; $∥u_{max}∥$ represents the maximum output of the controller; $k_{L}^{\left(k\right)}$ represents the potential field intensity generated by the *k*-th point on the trajectory *L*. τ(.) satisfies the following: τ(x)=1,x>0; otherwise, τ(x)=0. The function τ(·) is therefore an activation indicator: only the trajectory segment that contains the current leader position contributes to the leader controller. The gains $k_{L}^{\left(k\right)}$ determine how strongly UAV.1 tracks the desired velocity on each segment, and they can be increased when faster velocity correction is required or decreased when smoother motion is preferred.

### 4.2. Visual Attention Artificial Potential Field (Algorithm VAPF)

VAPF applies the visual attention rule directly to individual UAVs. After the selected neighbors are obtained, the controller separates them by distance so that attraction, velocity alignment, and repulsion are activated in different distance regions. Select attention ratio as AR, neighboring node $N_{i}$ according to the attention mechanism above, and divide $N_{i}$ into the following sets based on $∥x_{j}-x_{i}∥$.

(12) $$\begin{array}{c} N_{i}^{g}=\left\{j|j\in N_{i},∥x_{j}-x_{i}∥\in \left[R_{h}^{ji},R_{g}^{ji}\right]\right\},\\ N_{i}^{h}=\left\{j|j\in N_{i},∥x_{j}-x_{i}∥\in \left[R_{a}^{ji},R_{h}^{ji}\right]\right\},\\ N_{i}^{a}=\left\{j|j\in N_{i},∥x_{j}-x_{i}∥\in \left(0,R_{a}^{ji}\right]\right\}, \end{array}$$

where $N_{i}^{g}$ represents the set of neighboring nodes of UAV.*i* in the gravitational black-hole potential field; $N_{i}^{h}$ represents the set of neighboring nodes of UAV.*i* in the homogeneous potential field; and $N_{i}^{a}$ represents the set of neighboring nodes of UAV.*i* in the anti-gravity potential field. For UAV.*i*, i≠1, their motion models can be represented as

(13) $$\begin{array}{cc} \; & x_{i}\left[n+1\right]=x_{i}\left[n\right]+\min\left\{∥v_{i}\left[n\right]∥,∥v_{max}∥\right\}\frac{v_{i}\left[n\right]}{∥v_{i}\left[n\right]∥}\Delta t, \end{array}$$

(14) $$\begin{array}{cc} \; & v_{i}\left[n+1\right]=\gamma {\tilde{v}}_{i}\left[n+1\right]+\frac{\left(1-\gamma \right)}{\min\left\{n,n_{h}\right\}+1}\sum_{k=0}^{\min\left\{n,n_{h}\right\}}{\tilde{v}}_{i}\left[n-k\right], \end{array}$$

(15) $$\begin{array}{cc} \; & {\tilde{v}}_{i}\left[n+1\right]={\tilde{v}}_{i}\left[n\right]+\min\left\{∥u_{i}\left[n\right]∥,∥u_{max}∥\right\}\frac{u_{i}\left[n\right]}{∥u_{i}\left[n\right]∥}, \end{array}$$

(16) $$\begin{array}{cc} \; & u_{i}\left[n\right]=\left\{k_{1}f_{g}^{\left[n\right]}\left(i\right)+k_{2}f_{h}^{\left[n\right]}\left(i\right)+k_{3}f_{a}^{\left[n\right]}\left(i\right)\right\}, \end{array}$$

where $x_{i}\left[n\right]$ represents the position of UAV.*i*; $v_{i}\left[n\right]$ represents the speed of UAV.*i*; $\gamma \in \left[0,1\right]$ represents the filtering coefficient; $n_{h}$ represents the number of historical speed records; the denominator $\min\{n,n_{h}\}+1$ is the number of velocity records in the finite-history average; $k_{1},k_{2},k_{3}$ are non negative constants, and satisfy $k_{1}+k_{2}+k_{3}=1$; $f_{g}^{\left[n\right]}\left(i\right)$ represents the force acting on UAV.*i* in the gravitational black-hole potential field; $f_{h}^{\left[n\right]}\left(i\right)$ represents the force acting on UAV.*i* in the homogeneous potential field; $f_{a}^{\left[n\right]}\left(i\right)$ represents the force acting on UAV.*i* in the anti-gravity potential field; and $f_{g}^{\left[n\right]}\left(i\right),f_{h}^{\left[n\right]}\left(i\right),f_{a}^{\left[n\right]}\left(i\right)$ represent the following. The three weights $k_{1},k_{2},k_{3}$ allocate control authority among attraction, velocity alignment, and collision avoidance. In practice, $k_{3}$ should be large enough to make repulsion dominant at short range, while $k_{1}$ and $k_{2}$ are tuned to balance aggregation speed and velocity synchronization.

(17) $$\begin{array}{c} f_{g}^{\left[n\right]}\left(i\right)=\sum_{j\in N_{i}^{g}}\{-k_{b}^{ji}\left[-R_{b}^{ji}\frac{\Delta x_{ji}\left[n-1\right]}{∥\Delta x_{ji}\left[n-1\right]∥}+\Delta x_{ji}\left[n-1\right]\right]\\ \;\;\;*\tau \left(R_{b}^{ji}-∥\Delta x_{ji}\left[n-1\right]∥\right)+k_{g}^{ji}\Delta x_{ji}\left[n-1\right]\}, \end{array}$$

where $\Delta x_{ji}\left[n\right]=x_{j}\left[n\right]-x_{i}\left[n\right]$; $R_{g}^{ji}$ represents the radius of the gravitational black-hole potential field generated by UAV.*j* on UAV.*i*; $R_{h}^{ji}$ represents the radius of the homogeneous potential field generated by UAV.*j* on UAV.*i*; $R_{b}^{ji}$ represents the radius of the black-hole potential field generated by UAV.*j* on UAV.*i*; $k_{b}^{ji}$ represents the intensity coefficient of the black-hole potential field generated by UAV.*j* on UAV.*i*; and $k_{g}^{ji}$ represents the intensity coefficient of the gravitational black-hole potential field generated by UAV.*j* on UAV.*i*.

(18) $$\begin{array}{c} f_{h}^{\left[n\right]}\left(i\right)=\sum_{j\in N_{i}^{h}}k_{h}^{ji}\Delta v_{ji}\left[n-1\right], \end{array}$$

where $R_{a}^{ji}$ represents the radius of the anti-gravity potential field generated by UAV.*j* on UAV.*i*; $k_{h}^{ji}$ represents the intensity coefficient of the homogeneous potential field generated by UAV.*j* on UAV.*i*; and $\Delta v_{ji}\left[n\right]=v_{j}\left[n\right]-v_{i}\left[n\right]$.

(19) $$\begin{array}{cc} f_{a}^{\left[n\right]}\left(i\right)=\sum_{j\in N_{i}^{a}}\frac{\xi_{ji}\left[n-1\right]}{\Xi_{i}\left[n-1\right]} & \left\{-\frac{1}{R_{a}^{ji}∥\Delta x_{ji}\left[n-1\right]∥\Delta x_{ji}\left[n-1\right]}+\frac{1}{{\left(\Delta x_{ji}\left[n-1\right]\right)}^{3}}\right\}, \end{array}$$

where $\xi_{ji}\left[n\right]$ represents UAV.*j* for UAV.*i* attention coefficient; $\frac{\xi_{ji}\left[n\right]}{\Xi_{i}\left[n\right]}$ represents UAV.*j* for UAV.*i* attention weight. The calculation formula for $\Xi_{i}\left[n\right]$ is as follows

(20) $$\begin{array}{c} \Xi_{i}\left[n\right]=\sum_{j\in N_{i}^{a}}\xi_{ji}\left[n\right]\\ \;\;\;=\sum_{j\in N_{i}^{a}}k_{ji}^{a}\frac{\exp\left\{∥\Delta v_{ji}\left[n\right]∥\cos∠\left(\Delta v_{ji}\left[n\right],\Delta x_{ji}\left[n\right]\right)\right\}}{∥\Delta x_{ji}\left[n\right]∥+\varepsilon }. \end{array}$$

The attention weight of UAV.*i* regarding UAV.*j* is positively correlated with the relative velocity projected onto their line of sight and negatively correlated with their distance; $k_{a}^{ji}$ represents the intensity coefficient of the anti-gravity potential field generated by UAV.*j* on UAV.*i*; and ε>0 is a constant. The notation ∠(a,b) denotes the angle between two vectors, so $\cos∠(\Delta v_{ji},\Delta x_{ji})$ extracts the component of relative velocity along the line of sight. The angular part of visual relevance has already been handled by the zoom-lens selection rule, which removes targets hidden inside previously selected perceived cones. After this angular filtering, the remaining question is whether a selected neighbor needs a short-range response: a closer UAV is easier to observe and more directly related to collision avoidance, while a UAV with larger approaching velocity along the line of sight requires stronger short-range response. This is why the attention coefficient uses distance and line-of-sight relative velocity as the minimal first-order state variables. Other measurable neighbor cues, such as bearing angle, bearing-rate change, distance-change rate, acceleration, or state-estimation confidence, could also be considered in future sensing-level models. They are not included here because they would require additional measurement assumptions and tuning beyond the formation control layer considered in this paper. Here ε prevents division by zero when two UAVs are very close, and it also limits the numerical gain of the attention coefficient.

**Remark** **1.** *The attention coefficient should be interpreted as a lightweight relevance score rather than a complete visual perception model. It is consistent with the biological intuition that nearer and faster-approaching objects receive more attention, while keeping the controller implementable with relative position and velocity estimates.*

### 4.3. Cluster Visual Attention Potential Field (Algorithm CVAPF)

CVAPF is introduced because individual-level attention alone can still leave locally disordered trajectories after UAVs aggregate. The cluster version treats a group of nearby and kinematically compatible UAVs as one higher-level visual object, so neighbor selection is performed between clusters while local synchronization is maintained inside each cluster. The algorithm CVAPF extends the cooperation between UAVs to the cooperation between clusters. Algorithm CVAPF has the following advantages over the algorithm VAPF.

(1) Because the algorithm CVAPF has a cluster merging mechanism, the time for the formation to reach the steady state is shorter than that of the algorithm VAPF.
(2) After the formation reaches a steady state, the synchronization performance of formation is better than that of the algorithm VAPF, due to the two-layer communication architecture of the algorithm CVAPF.
(3) With cluster merging, the number of external neighbor objects processed by CVAPF can be reduced because several UAVs are treated as one cluster-level visual object.

The following will explain the methods for generating cluster behavior.

#### 4.3.1. Cluster Attention Neighbor Node Selection

**Figure 3:**
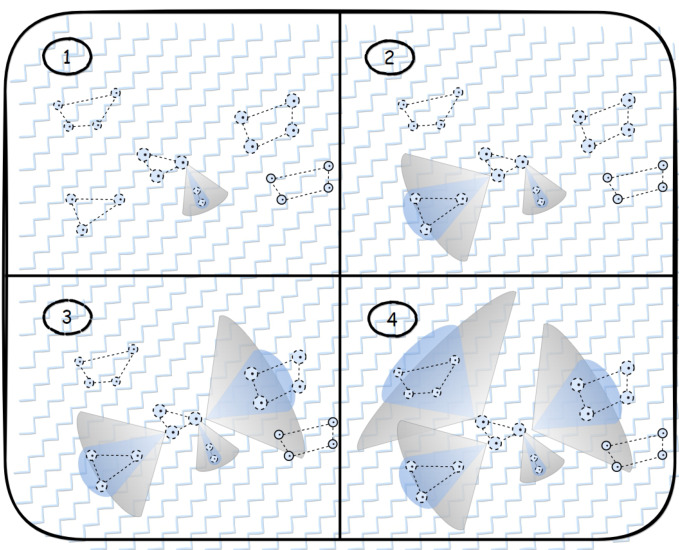
Cluster attention mechanism for selecting neighboring nodes. The numbers 1–4 indicate the cluster-neighbor selection order, light-blue sectors denote sensed angles, gray sectors denote perceived defocusing angles, and UAVs connected by dashed black lines belong to the same cluster.

Figure 3 illustrates how the individual zoom-lens rule in Figure 2 is lifted to the cluster level. Unlike BOIDS and Optimized-flocking, which select individual neighbors using local interaction rules, CVAPF first groups nearby UAVs and then applies visual attention to cluster-level objects. Each cluster is treated as a visual object whose angular span is determined by its boundary UAVs. After the nearest cluster is selected, its perceived cone masks angularly redundant clusters behind the same visual sector. This operation keeps the inter-cluster topology sparse while preserving the clusters that are geometrically most informative for aggregation (reference Figure 3). The distance from cluster J to cluster I is represented as

(21) $$\begin{array}{c} ∥\Delta X_{\mathrm{JI}}∥=\min∥x_{i}-x_{j}∥,i\in I,j\in J,\\ \;\;\;=∥x_{i}^{*}-x_{j}^{*}∥,i^{*}\in I,j^{*}\in J. \end{array}$$

(22) $$\begin{array}{c} \Delta V_{\mathrm{JI}}=v_{j}^{*}-v_{i}^{*}, \end{array}$$

where UAV.$i^{*}$ and UAV.$j^{*}$ are the two closest UAVs to Cluster I and Cluster J. For UAV-I(1∉I), it represents the set of UAV clusters. A single UAV is recorded as a cluster with a quantity of 1.

Step 1, select the cluster $J^{\left[1\right]}$ closest to cluster *I*.

(23) $$\begin{array}{c} J^{\left[1\right]}=\underset{J\in U}{arg min}∥\Delta X_{\mathrm{JI}}∥. \end{array}$$

The set of all clusters is denoted as *U*. $X_{I};X_{J}$ are the position of clusters I and J; Cluster I can receive information from cluster $J^{\left[1\right]}$ at this point, denoted as

(24) $$\begin{array}{c} N_{I}^{\left[1\right]}=J^{\left[1\right]}. \end{array}$$

The Perceived angle generated by I on $J^{\left[1\right]}$ is denoted as $\Theta_{{\mathrm{IJ}}^{\left[1\right]}}$, and the Sensed angle is denoted as $\theta_{{\mathrm{IJ}}^{\left[1\right]}}$. The cone corresponding to a Sensed angle is denoted by $C_{\theta_{{\mathrm{IJ}}^{\left[1\right]}}}$, and the cone corresponding to a Perceived angle is denoted by $C_{\Theta_{{\mathrm{IJ}}^{\left[1\right]}}}$. We have

(25) $$\begin{array}{c} \Theta_{{\mathrm{IJ}}^{\left[1\right]}}=AR_{C}\cdot \theta_{{\mathrm{IJ}}^{\left[1\right]}}, \end{array}$$

where $AR_{C}>0$ is the cluster-level attention ratio. A larger $AR_{C}$ masks a wider angular region after one cluster has been selected, so fewer external clusters are processed; a smaller $AR_{C}$ keeps more clusters and increases the amount of state information used by the controller.

(26) $$\begin{array}{c} \theta_{{\mathrm{IJ}}^{\left[1\right]}}=\max\left\{\arccos\left(\frac{\left(\Delta x_{ji^{*}},\Delta x_{j^{\prime }i^{*}}\right)}{∥\Delta x_{ji^{*}}∥∥\Delta x_{j^{\prime }i^{*}}∥}\right)+\arcsin\frac{r}{∥\Delta x_{ji^{*}}∥}+\arcsin\frac{r}{∥\Delta x_{j^{\prime }i^{*}}∥}\right\}, \end{array}$$

where $\Delta x_{ji^{*}}=x_{j}-x_{i^{*}},i^{*}\in I;j,j^{\prime }\in J^{\left[1\right]}$; *r* is the radius of the UAV,

(27) $$\begin{array}{c} x_{i^{*}}=arg\min_{x_{i}}\left\{∥x_{j}-x_{i}∥|j\in J^{\left[1\right]}\right\}. \end{array}$$

Step k, select the cluster $J^{\left[k\right]}$ closest to cluster *I*,

(28) $$\begin{array}{c} J^{\left[k\right]}=\underset{J\in U-{⋃}_{m=1}^{k-1}J^{\left[m\right]}}{arg min}∥\Delta X_{\mathrm{JI}}∥, \end{array}$$

by (26) and (27), we have $\theta_{{\mathrm{IJ}}^{\left[k\right]}}$. Here $C_{\Theta_{I}^{\left[k-1\right]}}$ denotes the union of perceived cones already selected before step *k*.

(29) $$N_{I}^{\left[k\right]}=\left\{\begin{array}{c} J^{\left[k\right]},C_{\theta_{{\mathrm{IJ}}^{\left[k\right]}}}⋂C_{\Theta_{I}^{\left[k-1\right]}}=\emptyset .\\ \emptyset ;otherwise. \end{array}\right.$$

The perceived cone union of cluster I is updated as

(30) $$\begin{array}{c} C_{\Theta_{I}^{\left[k\right]}}=\underset{J^{\left[k\right]}\in N_{I}^{\left[k\right]}}{⋃}C_{\Theta_{{\mathrm{IJ}}^{\left[k\right]}}}, \end{array}$$

The set of neighbors selected by cluster I can be represented as

(31) $$\begin{array}{c} N_{I}=\underset{k}{⋃}N_{I}^{\left[k\right]}. \end{array}$$

Internal communication of cluster I or cluster J, selecting $AR_{C}^{IN}$ as the attention ratio

$$N_{i}^{IN}=\left\{j|j\in N_{i}\cap I\right\}.$$

The intra-cluster ratio $AR_{C}^{IN}$ controls how many members of the same cluster are used for velocity alignment. In our design, external cluster links drive aggregation, whereas $N_{i}^{IN}$ provides local synchronization after members have merged into the same cluster.

**Remark** **2.** *The size of the inter-cluster topology changes dynamically with the number of clusters. When clusters merge, fewer external cluster-level objects need to be processed by the visual attention rule, while UAVs within the same cluster mainly use local links for velocity synchronization. This is the main difference between CVAPF and a fixed-radius neighbor rule: CVAPF does not process all visible UAVs independently after clustering, but separates sparse inter-cluster interaction from low-cost intra-cluster synchronization*.

#### 4.3.2. Cluster Motion Update

Cluster motion update converts selected cluster neighbors into control inputs applied through representative closest UAV pairs. The purpose is to make external cluster interactions sparse while still using intra-cluster velocity alignment to keep merged members moving coherently. Based on $∥\Delta X_{\mathrm{JI}}∥$, split the neighboring node $N_{I}$ into the following sets

(32) $$\begin{array}{cc} \; & N_{I}^{g}=\left\{J|J\in N_{I},∥\Delta X_{\mathrm{JI}}∥\in \left[R_{h}^{\mathrm{JI}},R_{g}^{\mathrm{JI}}\right]\right\},\\ \; & N_{I}^{h}=\left\{J|J\in N_{I},∥\Delta X_{\mathrm{JI}}∥\in \left[R_{a}^{\mathrm{JI}},R_{h}^{\mathrm{JI}}\right]\right\},\\ \; & N_{I}^{a}=\left\{J|J\in N_{I},∥\Delta X_{\mathrm{JI}}∥\in \left(0,R_{a}^{\mathrm{JI}}\right]\right\}. \end{array}$$

Here $R_{g}^{\mathrm{JI}}$, $R_{h}^{\mathrm{JI}}$, and $R_{a}^{\mathrm{JI}}$ are the cluster-level gravitational, homogeneous, and anti-gravity radii generated by cluster J for cluster I, respectively. They play the same role as $R_{g}^{ji}$, $R_{h}^{ji}$, and $R_{a}^{ji}$ in the individual-level VAPF model. The anti-gravity set is restricted to $\left(0,R_{a}^{\mathrm{JI}}\right]$ so that cluster-level repulsion is activated only in the short-range safety region.

For cluster UAV.*i*, i≠1, their motion models can be represented as

(33) $$\begin{array}{cc} {\tilde{v}}_{i}\left[n+1\right]={\tilde{v}}_{i}\left[n\right]+ & \alpha \sum_{j\in N_{i}^{IN}}\left(v_{j}\left[n\right]-v_{i}\left[n\right]\right)+\min\left\{∥U_{i}\left[n\right]∥,∥U_{max}∥\right\}\frac{U_{i}\left[n\right]}{∥U_{i}\left[n\right]∥}; \end{array}$$

(34) $$\begin{array}{c} U_{i}\left[n+1\right]=\left\{K_{1}F_{g}^{\left[n\right]}\left(i\right)+K_{2}F_{h}^{\left[n\right]}\left(i\right)+K_{3}F_{a}^{\left[n\right]}\left(i\right)\right\}, \end{array}$$

where α is the intra-cluster velocity alignment gain, and $∥U_{max}∥$ is the maximum allowed cluster-level control input; $K_{1},K_{2},K_{3}$ are non-negative constants, and satisfy $K_{1}+K_{2}+K_{3}=1$; $F_{g}\left(i\right)$ represents the force exerted by external clusters on UAV-*i* in the gravitational black-hole potential field; $F_{h}\left(i\right)$ represents the force exerted by external clusters on UAV-*i* in the homogeneous potential field; and $F_{a}\left(i\right)$ represents the force exerted by external clusters on UAV-*i* in the anti-gravity potential field. The constants $K_{1},K_{2},K_{3}$ have the same physical roles as $k_{1},k_{2},k_{3}$, but they are applied to interactions between clusters rather than interactions between individual UAVs. For clusters I and J, $F_{g}^{\left[n\right]}\left(i\right),F_{h}^{\left[n\right]}\left(i\right),F_{a}^{\left[n\right]}\left(i\right)$ represent the following.

$$\begin{array}{c} F_{g}^{\left[n\right]}\left(i\right)=0,i\neq i^{*}, \end{array}$$

(35) $$\begin{array}{cc} \; & F_{g}^{\left[n\right]}\left(i\right)=\sum_{J\in N_{I}^{g}}\{-k_{b}^{\mathrm{JI}}\left[-R_{b}^{\mathrm{JI}}\frac{\Delta X_{\mathrm{JI}}^{\left[n-1\right]}}{∥\Delta X_{\mathrm{JI}}^{\left[n-1\right]}∥}+\Delta X_{\mathrm{JI}}^{\left[n-1\right]}\right]\\ \; & \cdot \tau \left(R_{b}^{\mathrm{JI}}-∥\Delta X_{\mathrm{JI}}^{\left[n-1\right]}∥\right)+k_{g}^{\mathrm{JI}}\Delta X_{\mathrm{JI}}^{\left[n-1\right]}\},otherwise, \end{array}$$

where $R_{g}^{\mathrm{JI}}$ represents the radius of the gravitational black-hole potential field generated by UAV-J on UAV-I; $R_{h}^{\mathrm{JI}}$ represents the radius of the homogeneous potential field generated by UAV-J on UAV-I; $R_{b}^{\mathrm{JI}}$ represents the radius of the black-hole potential field generated by UAV.J on UAV.I; $k_{b}^{\mathrm{JI}}$ represents the intensity coefficient of the black-hole potential field generated by UAV.J on UAV.I; and $k_{g}^{\mathrm{JI}}$ represents the intensity coefficient of the gravitational black-hole potential field generated by UAV.J on UAV.I.

$$\begin{array}{cc} F_{h}^{\left[n\right]}\left(i\right)=0,i\neq & i^{*}, \end{array}$$

(36) $$\begin{array}{c} F_{h}^{\left[n\right]}\left(i\right)=-\sum_{J\in N_{I}^{h}}k_{h}^{\mathrm{JI}}\Delta V_{\mathrm{IJ}},otherwise, \end{array}$$

where $R_{a}^{\mathrm{JI}}$ represents the radius of the anti-gravity potential field generated by UAV-J on UAV-I; $k_{h}^{\mathrm{JI}}$ represents the intensity coefficient of the homogeneous potential field generated by UAV-J on UAV-I.

$$\begin{array}{cc} F_{a}^{\left[n\right]}\left(i\right)=0,i\neq & i^{*}, \end{array}$$

(37) $$\begin{array}{c} F_{a}^{\left[n\right]}\left(i\right)=\sum_{J\in N_{I}^{a}}\frac{\xi_{\mathrm{JI}}\left[n-1\right]}{\Xi_{I}\left[n-1\right]}\{-\frac{1}{R_{a}^{\mathrm{JI}}∥\Delta X_{\mathrm{JI}}^{\left[n-1\right]}∥\Delta X_{\mathrm{JI}}^{\left[n-1\right]}}+\frac{1}{{\left(\Delta X_{\mathrm{JI}}^{\left[n-1\right]}\right)}^{3}}\},otherwise, \end{array}$$

where $\xi_{\mathrm{JI}}$ represents UAV-J for UAV-I attention coefficient; $\frac{\xi_{\mathrm{JI}}}{\Xi_{I}}$ represents UAV-J for UAV-I attention weight. The calculation formula for $\Xi_{I}$ is as follows.

(38) $$\begin{array}{c} \Xi_{I}\left[n\right]=\sum_{J\in N_{I}^{a}}\xi_{\mathrm{JI}}\left[n\right]\\ \;\;\;=\sum_{J\in N_{I}^{a}}k_{\mathrm{JI}}^{a}\frac{\exp\left\{∥\Delta V_{\mathrm{JI}}^{\left[n-1\right]}∥\cos∠\left(\Delta V_{\mathrm{JI}}^{\left[n-1\right]},\Delta X_{\mathrm{JI}}^{\left[n-1\right]}\right)\right\}}{∥\Delta X_{\mathrm{JI}}^{\left[n-1\right]}∥+\varepsilon }, \end{array}$$

The cluster-level attention weight follows the same relevance logic as the individual-level coefficient: distance measures short-range interaction urgency, and $\cos∠(\Delta V_{\mathrm{JI}},\Delta X_{\mathrm{JI}})$ extracts whether the two closest representative UAVs are approaching along their line of sight. $k_{a}^{\mathrm{JI}}$ represents the intensity coefficient of the anti-gravity potential field generated by UAV-J on UAV-I. Thus, a cluster that is closer or approaching faster receives a larger repulsive weight, which is consistent with the purpose of avoiding close-range conflicts between clusters.

**Figure 4:**
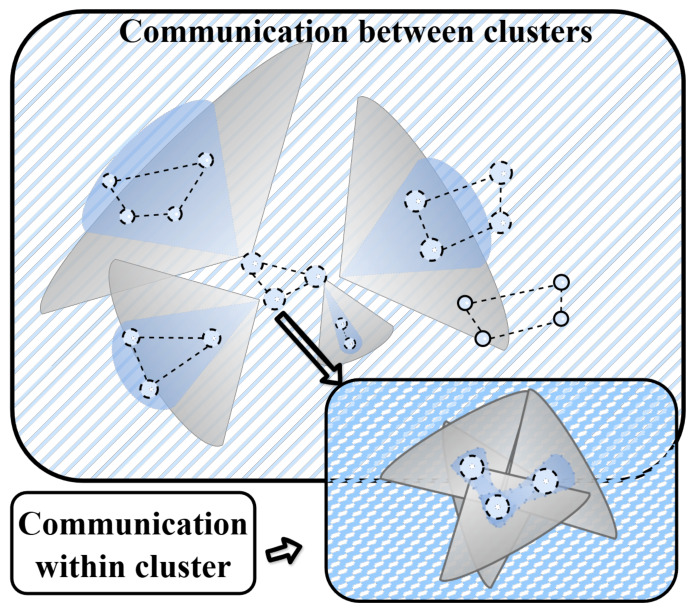
Dual layer communication architecture. Light-blue sectors denote sensed angles, gray sectors denote perceived defocusing angles, and UAVs connected by dashed black lines belong to the same cluster.

Figure 4 summarizes the data flow of CVAPF. The upper layer uses the cluster visual attention rule to choose only a small number of external clusters and applies potential-field forces through representative closest UAV pairs. The lower layer uses intra-cluster links to align velocities among UAVs that have already merged into the same cluster. Therefore, aggregation is controlled at the cluster level, while smooth local motion is controlled at the member level.

**Remark** **3.** *The dual layer communication architecture designed by the CVAPF algorithm is designed for distributed communication between and within clusters. Distributed communication between clusters is used to support aggregation between clusters, while distributed communication within clusters is used to support velocity consistency inside the cluster. In this way, CVAPF evaluates fewer external visual objects after merging while preserving local synchronization links among members of the same cluster*.

#### 4.3.3. Cluster Merging

Cluster merging changes the interaction topology, so the merge operation is made conservative. The following three conditions require spatial proximity, pairwise velocity compatibility, and internal velocity consistency before two clusters are fused. When groups approach each other, they will merge if they satisfy the following three conditions.

The merging rules are introduced to prevent two clusters from being fused before they are physically close and kinematically compatible.

Condition 1. The distance between clusters satisfies the following:

(39) $$\begin{array}{c} ∥\Delta X_{\mathrm{JI}}∥<R_{\mathrm{JI}}. \end{array}$$

Condition 2. The speed of the two closest UAVs between clusters should be sufficiently close.

(40) $$\begin{array}{c} 1-\frac{v_{i}^{*}v_{j}^{*}}{\max\left\{{∥v_{i}^{*}∥}^{2},{∥v_{j}^{*}∥}^{2}\right\}}\leq \epsilon_{a},i^{*}\in I,j^{*}\in J. \end{array}$$

Condition 3. The UAVs within each cluster have similar speeds.

(41) $$\begin{array}{cc} 1-\min_{i_{1},i_{2}\in I;j_{1},j_{2}\in J} & \{\frac{v_{i_{1}}v_{i_{2}}}{\max\left\{{∥v_{i_{1}}∥}^{2},{∥v_{i_{2}}∥}^{2}\right\}},\frac{v_{j_{1}}v_{j_{2}}}{\max\left\{{∥v_{j_{1}}∥}^{2},{∥v_{j_{2}}∥}^{2}\right\}}\}\leq \epsilon_{b}, \end{array}$$

$R_{\mathrm{JI}}$ represents the distance that UAV-I and UAV-J can merge, $\epsilon_{a},\epsilon_{b}$ are any small positive constants; and smaller $\epsilon_{a}$ and $\epsilon_{b}$ make merging stricter because they require stronger velocity similarity, while larger values allow earlier merging at the cost of possible transient disorder. When (39)–(41) are satisfied, we have

(42) $$\begin{array}{c} I:=I⋃J. \end{array}$$

**Remark** **4.** *The merging between clusters requires the two clusters to be close enough, and the speed between the two closest UAVs in the two clusters must be sufficiently similar. And each cluster needs to achieve autonomy before it can be merged, which means that the speed of all UAVs within the cluster must be highly similar before it can be satisfied. These conditions are all aimed at ensuring that cluster merging should be a gradual process. This process is similar to two trains that need to be docked; they need to be close enough in distance and speed. And the speed of each carriage in each train needs to be similar*.

### 4.4. Calculate the Boundary Conditions

This subsection gives geometric boundary conditions used for parameter selection. These conditions are practical constraints for avoiding close-range collision and maintaining local visual links under the stated assumptions, not a complete stability proof for arbitrary switching topologies.

The discussion is divided into two parts. First, a worst-case head-on encounter is used to define a safety margin for the anti-gravity field. Second, this margin is used to derive a sufficient angular condition for preserving local communication links inside a small three-UAV unit.

#### 4.4.1. Avoid Collision (Reference Figure 5)

Consider the most extreme situation: when two clusters are moving towards each other at maximum speed $∥v_{max}∥$ and their initial distance is $R_{a}^{\mathrm{JI}}$. We hope that they do not collide and the minimum distance can still maintain communication properties when constructing communication topologies. When we analyze the cluster as a whole, we do not consider the forces within the cluster. At this point, consider the cluster model as rigid movement (in reality, the position movement of each UAV within the cluster is not rigid movement. However, we only consider the two closest UAVs in the two clusters, so it does not affect it). Under this worst-case setting, the anti-gravity term is isolated because it is the component responsible for short-range separation.

(43) $$\begin{array}{cc} U_{I}\left[n+1\right] & =K_{3}\left\{\frac{1}{{\left(\Delta X_{\mathrm{IJ}}^{\left[n\right]}\right)}^{3}}+\frac{1}{R_{a}^{\mathrm{JI}}∥\Delta X_{\mathrm{IJ}}^{\left[n\right]}∥\Delta X_{\mathrm{IJ}}^{\left[n\right]}}\right\}, \end{array}$$

(44) $$\begin{array}{cc} U_{J}\left[n+1\right] & =-U_{I}\left[n+1\right]. \end{array}$$

We need to constrain clusters to avoid collisions

(45) $$\begin{array}{c} R_{safe}=\min_{I,J}R_{a}^{\mathrm{JI}}-\min\left\{∥X_{J}\left[k\right]-X_{I}\left[k\right]∥\right\}>0. \end{array}$$

In this constraint, $R_{safe}$ is the remaining distance margin after the strongest head-on repulsive interaction. Positive $R_{safe}$ means that the minimum simulated inter-cluster distance is still smaller than the activation radius $R_{a}^{\mathrm{JI}}$ by a nonzero safety margin, so the anti-gravity field can stop the two closest UAVs before collision.

**Figure 5:**
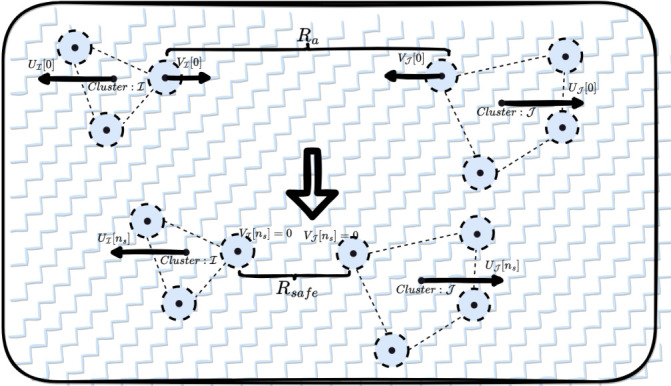
Cluster collision avoidance. Blue disks denote UAVs, and UAVs connected by dashed black lines belong to the same cluster. Solid black arrows denote velocity or control-input directions, and bracketed distances show the anti-gravity activation radius $R_{a}$ and the remaining safe distance $R_{safe}$.

#### 4.4.2. Maintain Communication

The communication maintenance condition links the safety margin to the visual attention parameter inside a cluster. The purpose is to prevent the perceived cone of one selected UAV from masking all other local candidates in the smallest connected unit.

**Figure 6:**
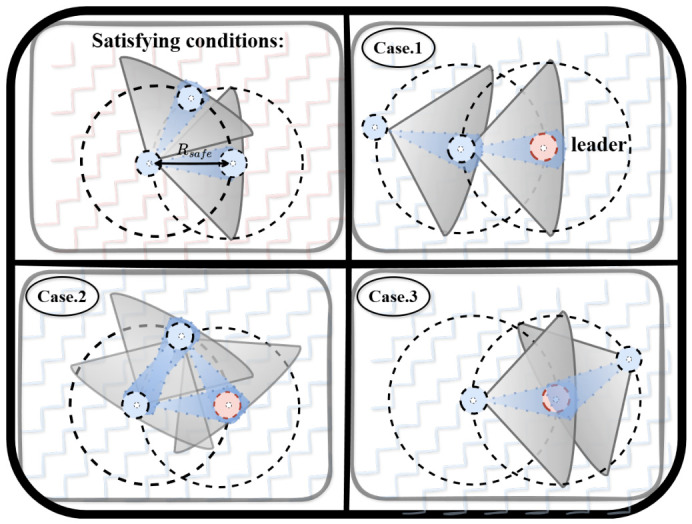
Communication maintenance condition in a three-UAV unit. Light-blue sectors denote sensed angles, gray sectors denote perceived defocusing angles. Dashed circular boundaries around UAVs denote the safety radius, UAVs with dashed borders are the focused UAVs retained by visual attention.

Using the safety margin above, we derive a sufficient geometric condition for maintaining local visual links in the communication topology generated within the cluster based on attention mechanism (refer to Figure 6). We consider three UAVs as a minimum unit, and they meet the following conditions. Here we consider one of the UAVs as the leader (not affect the generation of communication topology). Using the same cone notation at the UAV level, $C_{\theta_{ij}}$ is the sensed cone of UAV.*j* observed from UAV.*i*, and $C_{\Theta_{ij}}$ is the corresponding perceived cone. The condition below prevents the perceived cone of one selected UAV from covering the sensed cone of another candidate UAV, so at least two local visual links can be preserved in the three-UAV unit.

(46) $$\begin{array}{c} C_{\Theta_{ij}}⋂C_{\theta_{ij}}=\emptyset , \end{array}$$

Because the smallest strongly connected unit in Figure 6 is arranged with an angular separation of π/3, this non-overlap condition leads to the following sufficient bound:

(47) $$\begin{array}{c} AR_{C}^{IN}\cdot \arcsin\left(\frac{r}{R_{safe}}\right)+\arcsin\left(\frac{r}{R_{safe}}\right)\leq \frac{\pi }{3}. \end{array}$$

Within the smallest unit, at least one UAV can receive information from the other two UAVs. Moreover, at least one UAV in the formation can receive information from the leader, which provides a sufficient local condition for preserving a visual information path to the leader under the assumed geometry.

**Remark** **5.** *Equation (47) is a sufficient geometric condition for preserving local visual links in the three-UAV unit shown in Figure 6. Extending this unit by adding nearby UAVs gives a practical construction rule for local connectivity under the stated geometric assumptions, but it should not be interpreted as a complete proof for arbitrary switching topologies. This argument should be interpreted as a geometric sufficient-condition analysis for maintaining local visual links under the stated assumptions, rather than as a full graph-theoretic stability proof for arbitrary switching topologies. Equation (47) also gives a practical rule for parameter selection: increasing $AR_{C}^{IN}$ reduces redundant intra-cluster observations, but it must remain below the bound implied by r and $R_{safe}$ to avoid disconnecting the local topology*.

## 5. Simulation Results and Discussion

This section evaluates the proposed methods in simulation with emphasis on the scope of this paper: visual neighbor selection and redundancy suppression. The simulations are not intended to be a full robustness benchmark for sensor noise, communication delay, obstacle-rich environments, or multirotor dynamics. To demonstrate the effectiveness of the proposed method, we designed three simulation examples. Example 1 verifies the effectiveness of Algorithm 1 in formation flight. Example 2 verifies the effectiveness of Algorithm 2 in formation flight. Example 3 is designed specifically for the main focus of this paper: comparing the neighbor processing load and visual redundancy of different neighbor-selection rules. It does not aim to provide a full robustness, convergence, or flight-dynamics benchmark. We compare the advantages and disadvantages of the two algorithms. The effectiveness of the algorithm has been demonstrated through simulation using Python 3.8.5.


**Algorithm 1.** Leader Flight Planning + Algorithm VAPF**Require:** Position $x_{i}$ and speed $v_{i}$ of each UAV.
**Ensure:** Formation behavior evaluated under the proposed VAPF update. 
  1: Initialization $r,AR,n_{l},{\left\{k_{L}^{k}\right\}}_{k=1}^{n_{l}+1},{\left\{v_{L}^{k}\right\}}_{k=1}^{n_{l}+1},{\left\{l^{\left(k\right)}\right\}}_{k=1}^{n_{l}+1}$, $∥v_{max}∥,∥u_{max}∥,\gamma ,n_{h},k_{1},k_{2},k_{3},$$R_{g}^{ji},R_{h}^{ji},R_{b}^{ji},R_{a}^{ji},$$k_{g}^{ji}$, $k_{h}^{ji},k_{b}^{ji}$, $k_{a}^{ji},\varepsilon .$
  2: At the n+1-th moment
  3. Initialization $x_{i}\left[n\right],v_{i}\left[n\right],\sum_{k=0}^{\min\left\{n,n_{h}\right\}}{\tilde{v}}_{i}\left[n-k\right].$
  4: Flight planning for leader (UAV.1).
  5: Calculate $u_{1}\left[n+1\right]\Rightarrow v_{1}\left[n+1\right]\Rightarrow x_{1}\left[n+1\right]$
  6: **for** UAV.*i*; i≠1 **do**
  7:        Using visual attention mechanism to select neighbor $N_{i}$.
  8:        Divide $N_{i}$ into $N_{i}^{g}$, $N_{i}^{h}$, and $N_{i}^{a}$ based on distance.
  9:        Calculate $f_{g}^{\left[n\right]}\left(i\right),f_{h}^{\left[n\right]}\left(i\right),f_{a}^{\left[n\right]}\left(i\right)\Rightarrow u_{i}\left[n\right]\Rightarrow {\tilde{v}}_{i}\left[n+1\right]\Rightarrow v_{i}\left[n+1\right]\Rightarrow x_{i}\left[n+1\right]$.
10: **end for**
**Algorithm 2.** Leader Flight Planning + Algorithm CVAPF**Require:** Position $x_{i}$ and speed $v_{i}$ of each UAV.
**Ensure:** Formation behavior evaluated under the proposed CVAPF update.
  1: Initialization $r,AR_{C},AR_{C}^{IN},n_{l},{\left\{k_{L}^{k}\right\}}_{k=1}^{n_{l}+1},{\left\{v_{L}^{k}\right\}}_{k=1}^{n_{l}+1},{\left\{l^{\left(k\right)}\right\}}_{k=1}^{n_{l}+1},∥v_{max}∥,∥u_{max}∥,\gamma ,n_{h},$$\alpha ,∥U_{max}∥,K_{1},K_{2},K_{3},R_{g}^{\mathrm{JI}},R_{h}^{\mathrm{JI}},R_{b}^{\mathrm{JI}},R_{a}^{\mathrm{JI}},$$k_{g}^{\mathrm{JI}}$, $k_{h}^{\mathrm{JI}},k_{b}^{\mathrm{JI}}$, $k_{a}^{\mathrm{JI}},\varepsilon ,R_{\mathrm{JI}},\epsilon_{a},\epsilon_{b}$
  2: At the n+1-th moment
  3: Initialization $x_{i}\left[n\right],v_{i}\left[n\right],\sum_{k=0}^{\min\left\{n,n_{h}\right\}}{\tilde{v}}_{i}\left[n-k\right].$
  4: Flight planning for leader (UAV.1).
  5: Calculate $u_{1}\left[n+1\right]\Rightarrow v_{1}\left[n+1\right]\Rightarrow x_{1}\left[n+1\right]$
  6: **for** UAV.*i*; i≠1 **do**
  7:      **if** Cluster UAV-I meets merging conditions (41) **then**
  8:            **for**J in *U* **do**
  9:                  **if** Cluster UAV-I and UAV-J meet condition (39)and (40) **then**
10:                        Cluster UAV-I and UAV-J merge to form a new cluster UAV-$I_{new}$
11:                  **end if**
12:            **end for**
13:      **end if**
14:      Using cluster visual attention mechanism to select neighbor $N_{I}$, and using visual attention mechanism to select neighbor $N_{i}^{IN}$.
15:      Divide $N_{I}$ into $N_{I}^{g}$, $N_{I}^{h}$, and $N_{I}^{a}$ based on $∥\Delta X_{\mathrm{JI}}∥$.
16:      Calculate $F_{g}^{\left[n\right]}\left(i\right),F_{h}^{\left[n\right]}\left(i\right),F_{a}^{\left[n\right]}\left(i\right)\Rightarrow U_{i}\left[n\right]\Rightarrow {\tilde{v}}_{i}\left[n+1\right]\Rightarrow v_{i}\left[n+1\right]\Rightarrow x_{i}\left[n+1\right]$.
17: **end for**


The simulation setup is reported before the examples so that the parameter choices are explicit. Table 1 and Table 2 give the leader settings and the common initial follower states used in Examples 1 and 2. In leader flight planning, the initial parameters we set refer to Table 1. These parameters will be used in the following two examples.

The parameters used in the following examples are selected according to their physical roles rather than by exhaustive optimization. The interaction radii follow the order $0<r<R_{a}<R_{h}<R_{b}<R_{g}$: $R_{a}$ defines the short-range safety region, $R_{h}$ marks the velocity alignment region, $R_{b}$ separates shaped intermediate attraction from long-range attraction, and $R_{g}$ is the largest distance at which a selected neighbor affects the APF controller. The same ordering principle is used for cluster-level interaction radii. The weights $k_{1},k_{2},k_{3}$ and $K_{1},K_{2},K_{3}$ are normalized to keep the input scale bounded, with the repulsive weight chosen largest so that collision avoidance dominates at short range. The filtering parameters γ and $n_{h}$ trade response speed for smoothness, while the merging thresholds $\epsilon_{a}$ and $\epsilon_{b}$ determine how strictly clusters must match velocities before fusion.

The initial speed of the remaining UAVs is 0, and their initial positions refer to Table 2.

**Example** **1.** *Algorithm 1 (leader flight planning + algorithm VAPF). In this example, we consider seven UAVs that rely on visual attention mechanisms to establish communication topologies and transmit state information. The unique parameter AR=4,r=1 used to construct communication topology. The various parameters in the motion of the UAVs are $\gamma =0.9;n_{h}=5;\varepsilon =1$; $k_{1}=0.2;k_{2}=0.2;k_{3}=0.6$; $R_{a}^{ji}=10,R_{h}^{ji}=40,R_{b}^{ji}=55,R_{g}^{ji}=200,k_{g}^{ji}=k_{b}^{ji}=k_{h}^{ji}=k_{a}^{ji}=1$.*

**Figure 7:**
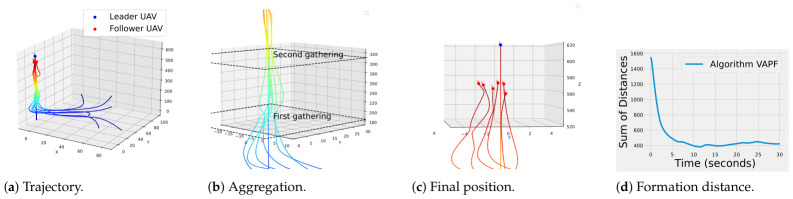
Algorithm VAPF simulation results. The color of the trajectory is mapped to time.

Figure 7 shows the experimental results of Algorithm 1 after simulation. Among them, Figure 7a represents the flight trajectory of the formation starting from its initial position within 30 s, and the color of the trajectory is mapped to time. The trajectory indicates that Algorithm 1 produces aggregation behavior in the tested scenario. However, as the formations approached each other, each UAV seemed to become hasty, causing the UAVs to repeatedly move from dispersion to aggregation, as shown in Figure 7b. In this simulation, Algorithm 1 shows strong aggregation behavior and no inter-UAV collision is observed. From a local perspective, the trajectory of the follower UAV is disorderly, as shown in Figure 7c.

**Example** **2.** *Algorithm 2 (leader flight planning + algorithm CVAPF). In this example, the attention ratio of establishing a communication topology between clusters is $AR_{C}=3,r=1$, and the attention ratio of establishing a communication topology between UAVs within a cluster is $AR_{C}^{IN}=2$. The parameters for cluster motion and merging are $\gamma =0.9;n_{h}=5;\varepsilon =1;\alpha =0.5$, $K_{1}=0.2;K_{2}=0.2;K_{3}=0.6$; $R_{a}^{\mathrm{JI}}=10,R_{h}^{\mathrm{JI}}=40,R_{b}^{\mathrm{JI}}=55,R_{g}^{\mathrm{JI}}=200$, $k_{g}^{\mathrm{JI}}=k_{b}^{\mathrm{JI}}=k_{h}^{\mathrm{JI}}=k_{a}^{\mathrm{JI}}=1$; $R_{\mathrm{JI}}=40;\epsilon_{a}=0.9;\epsilon_{b}=0.95.$*

**Figure 8:**
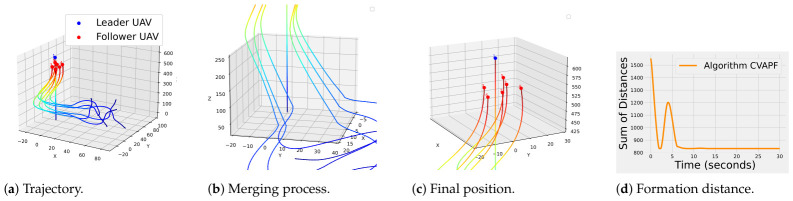
Algorithm CVAPF. The color of the trajectory is mapped to time.

Figure 8 shows the experimental results of Algorithm 2 after simulation. Among them, Figure 8a represents the flight trajectory of the formation starting from its initial position within 30 s, and the color of the trajectory is mapped to time. The trajectory shows that Algorithm 2 improves aggregation and synchronization in the tested scenario. As formation mergers occur, the trajectory of the formation becomes increasingly synchronized, as shown in Figure 8b. From a local perspective, the formation consistency driven by Algorithm 2 is more orderly than the VAPF trajectory in Figure 7c, as shown in Figure 8c.

**Remark** **6.** *Algorithm 1 has better aggregation performance than Algorithm 2 (refer to Figure 7d and Figure 8d). The sum of distances $\frac{1}{2}\sum_{i=1}^{n}\sum_{j=1}^{n}∥x_{i}-x_{j}∥$ for the formation in Algorithm 1 is approximately 400, which is only half of Algorithm 2. In the tested scenario, Algorithm 1 produces a more compact final aggregation, while Algorithm 2 produces more synchronized trajectories because cluster merging reduces repeated external neighbor processing and strengthens intra-cluster velocity alignment. Due to the setting of the condition (41) for cluster merging, the distance between UAVs is far greater than that of Algorithm 1*.

**Example** **3.** *Comparative neighbor processing experiment*.

To address whether visual attention provides a measurable advantage over standard neighbor-selection rules, we further test a redundant sensing scene with 45 UAVs in the same spatial configuration. The compared methods are baseline BOIDS [16], which processes all UAVs inside a fixed sensing radius; baseline Optimized-flocking [19], which uses a shorter effective interaction radius motivated by the optimized autonomous-drone flocking model; VAPF; and CVAPF. This experiment evaluates only the neighbor-selection topology, so all methods are compared under the same positions, velocities, and sensing scene.

**Figure 9:**
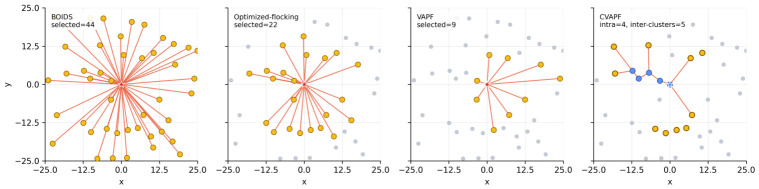
Neighbor selection in a redundant UAV sensing scene. The star denotes the inspected UAV or reference cluster, yellow points denote selected neighbors or selected neighbor clusters, gray points denote unselected UAVs, red lines denote processed neighbor links, and blue points denote intra-cluster selected UAVs.

The qualitative comparison in Figure 9 shows that the two baseline methods retain many angularly overlapping neighbors, whereas VAPF and CVAPF suppress redundant neighbors in the same visual sector. The quantitative results in Table 3 support the same conclusion. The average processed neighbors per UAV decrease from 43.38 in BOIDS and 15.20 in the Optimized-flocking baseline to 6.31 in VAPF and 3.60 in CVAPF. In this experiment, a redundant neighbor means a farther UAV whose visual cone is occluded by a nearer UAV in the same viewing direction. Such an occluded UAV does not provide additional effective visual information to the reference UAV, but including it as a neighbor still increases the controller’s state-processing load. Therefore, the redundancy ratio is computed by scanning the candidate neighbors from near to far and counting the farther UAVs that fall inside cones already occupied by nearer UAVs. This test gives redundancy ratios of 0.318 for BOIDS and 0.273 for Optimized-flocking, while VAPF and CVAPF have no additional occlusion redundancy after attention-based selection. Therefore, under the same sensing scene, the proposed visual attention topology preserves the APF-driven formation behavior observed in Examples 1 and 2 while substantially reducing redundant neighbor processing.

To clarify the influence of the attention ratio, we further vary AR in the same redundant sensing scene and keep all other settings unchanged. This analysis is not intended as a full controller sensitivity study; it isolates the neighbor-selection parameter that directly controls angular masking. A larger AR creates a wider perceived cone after a target is selected, so more angularly redundant candidates are suppressed and the average number of processed neighbors decreases.

Table 4 shows the expected monotonic trend. For VAPF, the average processed neighbors decrease from 22.73 to 4.73 as AR increases from 1 to 5. For CVAPF, the value decreases from 7.96 to 3.29 because cluster merging already suppresses many external objects before the attention ratio is varied. These results explain the practical role of AR: it trades retained visual information for lower neighbor processing load, and it should be chosen together with the communication maintenance bound in (47).

## 6. Conclusions

Considering formation control in complex environments, this paper proposes two visual-attention-based neighbor-selection algorithms based on a visual attention mechanism to generate communication topology, namely VAPF and CVAPF. The VAPF algorithm uses an individual-level zoom-lens rule to select informative UAV-level neighbors before APF control evaluation, which supports aggregation in the tested scenario. The CVAPF algorithm extends this neighbor-selection idea to the cluster level through a dual layer communication architecture and cluster merging. It reduces repeated external-neighbor processing and improves the synchronization behavior observed in the simulations. The simulations show that the proposed neighbor-selection algorithms preserve APF-driven formation behavior while reducing average processed neighbors to 6.31 and 3.60, compared with 43.38 for BOIDS and 15.20 for Optimized-flocking. Future work will study robustness under more realistic sensing and flight conditions.

## Figures and Tables

**Table 1:** Leader flight planning simulation parameters.

Items	Simulation Parameters
Initial Position	$x_{1}\left[0\right]={\left(0,0,20\right)}^{T}$
Initial Speed	$v_{1}\left[0\right]={\left(0,0,20\right)}^{T}$
Maximum Speed	$∥v_{max}∥=20$
Maximum Input	$∥u_{max}∥=40$
Divide Quantity	$n_{l}=580$
Potential Field Intensity Coefficient	${\left\{k_{L}^{\left(k+1\right)}\right\}}_{k=1}^{n_{l}}=1$
Preset Expected Speed	${\left\{v_{L}^{\left(k+1\right)}\right\}}_{k=1}^{n_{l}}={\left(0,0,20\right)}^{T}$
Division Point Position	${\left\{l^{\left(k+1\right)}\right\}}_{k=0}^{n_{l}}={\left(0,0,20+k\right)}^{T}$

**Table 2:** Initial position of the follower UAVs.

Items	Simulation Parameters
Initial Position of UAV.2	$x_{2}\left[0\right]={\left(37.45,95.07,73.19\right)}^{T}$
Initial position of UAV.3	$x_{3}\left[0\right]={\left(5.81,86.62,60.11\right)}^{T}$
Initial position of UAV.4	$x_{4}\left[0\right]={\left(83.24,21.23,18.18\right)}^{T}$
Initial position of UAV.5	$x_{5}\left[0\right]={\left(43.19,29.12,61.18\right)}^{T}$
Initial position of UAV.6	$x_{6}\left[0\right]={\left(45.60,78.51,19.96\right)}^{T}$
Initial position of UAV.7	$x_{7}\left[0\right]={\left(60.75,17.05,6.51\right)}^{T}$

**Table 3:** Quantitative comparison of neighbor processing load and redundancy ratio in the redundant sensing scene.

Method	Avg. Neighbors	Redundant Ratio
Baseline BOIDS	43.38	0.318
Baseline Optimized-flocking	15.20	0.273
VAPF	6.31	0.000
CVAPF	3.60	0.000

**Table 4:** Average processed neighbors under different attention ratios in the redundant sensing scene.

Method	AR=1	AR=2	AR=3	AR=4	AR=5
VAPF	22.73	13.80	8.89	6.31	4.73
CVAPF	7.96	5.22	4.27	3.60	3.29

## Data Availability

Data is contained within the article.
